# 3D technology to measure dental arches and create a template for lingual brackets technique

**DOI:** 10.1590/2177-6709.26.3.e2119234.oar

**Published:** 2021-06-30

**Authors:** Silvana Allegrini KAIRALLA, Mario CAPPELLETTE, Leandro VELASCO, Leila Soares FERREIRA, Shirley Shizue Nagata PIGNATARI

**Affiliations:** 1 Universidade Federal de São Paulo, Faculdade de Medicina, Departamento de Otorrinolaringologia e Cirurgia de Cabeça e Pescoço (São Paulo/SP, Brazil).

**Keywords:** Dental arch, Anatomy, Orthodontics

## Abstract

**Objective:**

This study aims at identifying anatomical dimensions of dental arches, based on landmarks currently used in the lingual orthodontic technique, and create an archwire form template to be used in orthodontic clinics.

**Methods:**

Maxillary and mandibular dental casts of 140 Caucasian individuals with natural and normal occlusion were digitized (3D), and the images were analyzed with Delcam Power Shape^TM^ 2010 software. The dental arch shapes and sizes were obtained from 14 landmarks selected on the lingual surface of the teeth. Points and segments defined by the software were used to create an archwire form template.

**Results:**

Various dental arch patterns were found for both maxilla and mandible. The smallest sizes were found in females, and the largest were found in male subjects. Six categories were defined for each gender, three for the maxilla and three for the mandible (Small, Medium and Large). A template was created with eighteen anatomic lingual archwire designs, nine for the maxilla and nine for the mandible, for both genders.

**Conclusions:**

Landmarks evaluated in this study showed dental arch differences between genders. This information enables making orthodontic lingual archwires that are more compatible with the anatomical forms and sizes of the maxilla and mandible. The findings also allowed the creation of a template for an anatomic lingual metallic archwire form to be used in the lingual technique.

## RESUMO

**Objetivo:**

O presente estudo tem como objetivo encontrar as formas anatômicas e dimensões das arcadas dentárias com base em pontos de referência utilizados na técnica ortodôntica lingual, e criar um diagrama com um maior número de arcos metálicos para serem utilizados na clínica ortodôntica.

**Métodos:**

140 modelos de indivíduos caucasianos com oclusão normal e natural foram digitalizados (3D) e as imagens, analisadas com o *software* Delcam Power Shape^TM^ 2010. A determinação das formas e tamanhos das arcadas dentárias foi obtida a partir de 14 pontos selecionados na superfície lingual dos dentes. Outros pontos e segmentos foram utilizados, com o auxílio do *software,* para definir um diagrama.

**Resultados:**

Foram encontrados diferentes tamanhos de arcadas dentárias linguais, tanto para a maxila quanto para a mandíbula. Os menores tamanhos foram os femininos, e os maiores, os masculinos. Definiram-se seis tamanhos para cada sexo, sendo três para a maxila e três para a mandíbula, nomeados como P, M e G. Foi criado um diagrama com dezoito desenhos de arcos linguais anatômicos, nove para a maxila e nove para a mandíbula, para ambos os sexos.

**Conclusões:**

A posição dos pontos de referência nesse estudo evidenciou diferenças entre os sexos, o que permitiu a criação de arcos mais compatíveis com as formas e dimensões anatômicas da maxila e mandíbula. A diferença entre os tamanhos das arcadas dentárias linguais possibilitou a criação de um diagrama com formas de arcos metálicos linguais anatômicos para serem utilizados na técnica lingual, para auxiliar o profissional a criar os seus próprios arcos.

## INTRODUCTION

In the late 1970s, lingual orthodontics was introduced as a result of conventional appliances bonding to the lingual surfaces of the teeth.^1^ ^,^ ^2^ The first scientific work describing brackets and the mushroom-shape of the lingual archwire was published in 1979.^3^

Striking differences between the lingual and buccal techniques are observed,^4^ such as the archwire form used.^5^ However, few studies^6^ ^-^ ^9^ have been published attempting to determine the dental arch form in the lingual technique. 

Many authors have used cusp tips to outline the archwire forms,^10^ ^,^ ^11^ ^,^ ^12^ while others used the medial landmarks of the crowns from a buccal perspective on the anterior and posterior teeth as references,^13^ as well as lingual and occlusal landmarks, or in the long axial axis of the teeth.^7^ Moreover, others researchers^14^ ^,^ ^15^ used landmarks on the lingual surfaces closer to the gingival third because this site showed the smallest difference between the lingual surfaces of canines and premolars.^16^

Despite the fact that there are several ways to define dental arch forms, in the lingual technique, the inter-canine distances vary substantially,^16^ making it difficult to determine how many sizes of mushroom-shaped lingual archwires might exist.^3^ Therefore, some authors developed the straight-wire concept in lingual orthodontics, seeking to streamline the work of the professional.^16^ ^,^ ^17^ They also proposed^18^ that brackets should be bonded with auxiliary blades in order to enable the use of archwires without curvatures, whereas other authors^14^ ^,^ ^15^ devised a more square-shaped archwire, allowing use of a lingual straight archwire. 

A previous study analyzing the shapes and dimensions of dental arches in digital 3D models for the use in lingual straight-wire technique showed that more cervical archwire setting promotes smaller inter-bracket distance.^15^

Although this strategy allowed reduction of the typical insets and offsets of the dental arch lingual surfaces, it hampers orthodontic mechanics in certain movements^19^ ^,^ ^20^ and may cause gingival inflammation.^21^ Furthermore, a specific assembly with resin pads is required to compensate the distance between the lingual surface and the base of the bracket.^4^ ^,^ ^5^ In areas where compensations are made, the brackets advance more into the space occupied by the tongue, resulting in patient discomfort.^21^ Furthermore, low bracket profile enables less invasion to the lingual space, therefore providing better adaptation for the patient in terms of speech and comfort.^22^

In this context, the present study evaluated the dental arch shapes and sizes that are formed when the brackets are placed farther from the cervical margin of the teeth,^15^ in region that keep the concave and convex form in the lingual surface, but more distant from the cervical area, to avoid gingival inflammation.

The forms and sizes of archwires for the lingual technique were also defined, and a template was created. It is believed that there is a difference in dental arch forms between genders. Orthodontists should benefit from different sizes and shapes of archwires to perform treatments, and not be limited to a number of prefabricated archwires that are usually dictated by wire and bracket manufacturers. Using the template, the professional may choose the appropriate archwire form. 

## MATERIAL AND METHODS

This study is an analytical observational research of patient records from Faculty of Health, UMESP, São Bernardo do Campo, SP, Brazil. The protocol of this study was approved by the Ethics Committee from, Federal University of São Paulo - UNIFESP, number 0388/2016.

The sample included maxillary and mandibular dental casts of 70 Caucasian Brazilian individuals (28 men and 42 women), minimum 15.0 years old and maximum of 21.3 years old (average 16.4 ±1.3 years old). 

Sample inclusion criteria were defined as follows: cast models from individuals with normal occlusion and no odontogenic abnormalities; complete full dentition, except for third molars; and all permanent teeth in occlusion according to the following keys for occlusion of Andrews :^23^ Angle Class I molar relationship, angulation and inclination of the crowns (considering the long axis of the teeth) and the flat curve of spee.^23^ Rotations of up to 3 degrees and diastema up to 0.5 mm were accepted.

Sample exclusion criteria were: odontogenic abnormalities, incomplete dental eruption, and presence of erupted third molars. 

Seventy pairs of cast models were digitized with a 3D Dental Wings™ scanner (model DW5-140, Montreal, Quebec, Canada). Images were analyzed by Delcam Power SHAPE™ software (2010, Birmingham, UK). 

To standardize the position of the models and to avoid measurement distortions, landmarks were set on the canine’s incisal and on the first molar’s mesiobuccal cusp,^10^ creating a trapezoid form and a grid of coordinates in the X, Y, and Z axes, allowing the rotation of the models in several positions, without changing the proportion of the measures executed during the study.^15^

### DETERMINING THE LANDMARKS AND DEFINING THE SHAPE AND SIZE OF THE ARCH

Lingual surface landmarks were determined with the Delcam Power SHAPE^™^ 2010 software program. The landmarks chosen represented the location where the brackets would be placed on the lingual surface of each tooth, and where the archwires would pass inside the bracket slot.

The location of the points was defined as follows: In the maxillary and mandibular anterior teeth, the reference point was located at the intersection of the line passing through the long axis of the tooth in the vertical plane and the line passing through the center of the clinical crown of the horizontal axis (the deepest point of the concavity of palatal surface). In the maxillary and mandibular premolars, the reference point was also located at the intersection of the line passing through the long axis of the tooth in the vertical plane and the line passing through the middle third of the lingual surface (the most prominent convex point of the lingual surface). In the first and second upper and lower molars, the reference point was located in the intersection of the line that passes the long axis of the mesio-lingual cusp in the vertical plane and the line passing through the middle third of the lingual surface (most prominent point of the lingual surface of these teeth). 

The model was rotated on the computer screen in a way that the lingual surfaces could be seen with a frontal view and the operator could define and locate where the marked landmarks should be placed.

**Figure 1:**
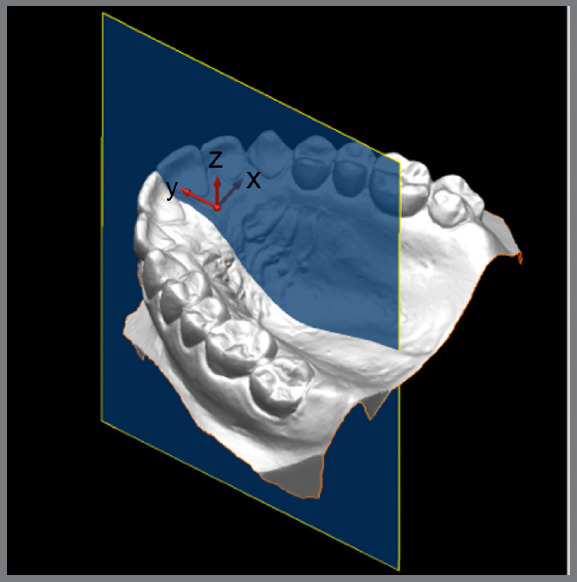
Digitalized model, with the X, Y, and Z coordinates.

By means of the X, Y, and Z coordinates, two planes were established. The X and Y axes established the horizontal plane, whereas the Y and Z axes established the vertical plane, corresponding to the median sagittal plane that passes between the central incisors and divides the model into two halves, left and right (Fig 1).

**Figure 2:**
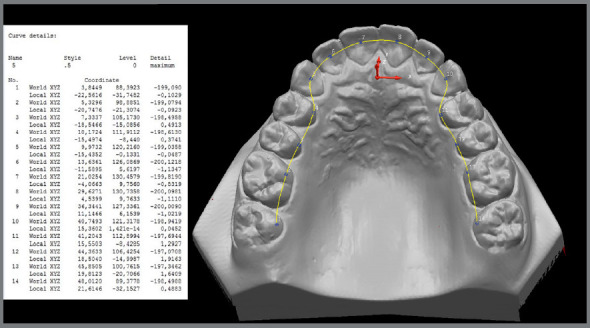
Image obtained from Delcam Power SHAPE™ 2010 software model, with points determined on the lingual surface and arch shape formed with measurements obtained from each key-point related to the coordinates (X, Y, and Z).

The fourteen united landmarks defined the curvature and shape of the dental lingual archwire^11^ using Delcam Power SHAPE™ 2010 tools (Fig 2).

The previously chosen points were plotted in the XY plane, such that the Z axis was reset, and it was possible to obtain the XY coordinates of each landmark (Fig 2).

For determining fourteen measurements (ten horizontal and four vertical),^10^ ^,^ ^15^ Delcam Power SHAPE™ 2010 program was used as well.

The horizontal measurements corresponded to the distances between the landmarks chosen in the molars, premolars and canines in relation to the Y axis; and the vertical measurements, to the central and lateral incisors in relation to the X axis (Fig 2).

The program provided linear measurements, in millimeters, of the coordinates of the points in relation to the work plane, indicating a satisfactory degree of accuracy (Fig 2).

### DETERMINING ARCHWIRE TEMPLATE

**Figure 3:**
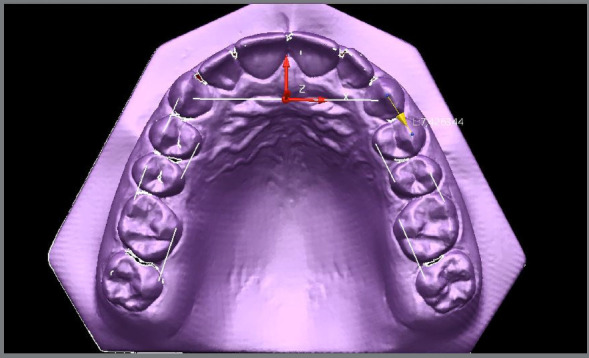
Eight straight wire segments defined by the landmarks and cuspid tips.

For the construction of the archwire template, the Delcam Power SHAPE™ 2010 program was used in the previously digitized models. Points were created on the cusp tips of the canines and first left and right premolars, and then points on the cusp tips of the second premolars and left and right first molars, and these points were united two-by-two, forming small straight line segments. Previously defined points were also used on the lingual surface of the 3D model of the first and second left and right molars, and the first and second left and right premolars, which were joined two-by-two, respectively, forming small straight line segments (Fig 3). 

**Figure 4:**
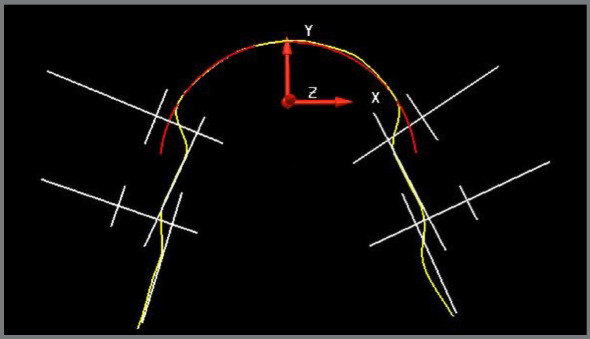
Lines and curves to define the archwire shapes for the template.

Perpendicular segments were drawn to the straight line segments previously created, and then the model was removed from the computer screen. An extension of all the lines was made until they crossed each other. The initial 3D model was reinserted, and perpendicular line extensions were found to pass through the interdental embrasure ridge of the respective teeth in question. That was also done in the mandibular model, and the measurements obtained were all flattened in the Z axis, so that a flat archwire could be constructed, because the models were in a three-dimensional space. At this stage, a curve (red line in Fig 4) was created from the point of the left canine to the point of the right canine, passing through a point between the central incisors, as the anterior limit of the curvature. 

**Figure 5:**
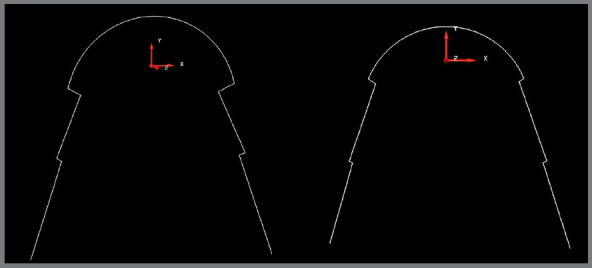
Maxillary and mandibular dental arches obtained by the Delcam Power SHAPE™ 2010 software.

The program has a tool in the form of scissors that allowed for all the extensions to be cut, thereby obtaining the final designs of the maxillary and mandibular archwires of each of the models (Fig 5). The templates with eighteen archwires, nine maxillary and nine mandibular, were built from the coordinates (X and Y) of the eleven points, which defined the design of the metallic archwires mentioned in Figure 5.

Percentiles 5, 15, 25, 40, 50 (medium), 60, 75, 85 and 95 were taken from the coordinates (X and Y) measurements of each point, and then the mean between both sides was symmetrical.

**Figure 6:**
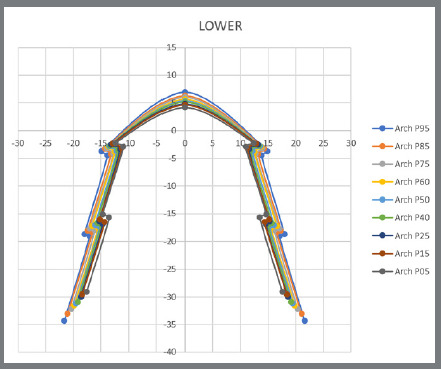
Illustration of the archwires’ points and the percentiles of the mandible.

**Figure 7:**
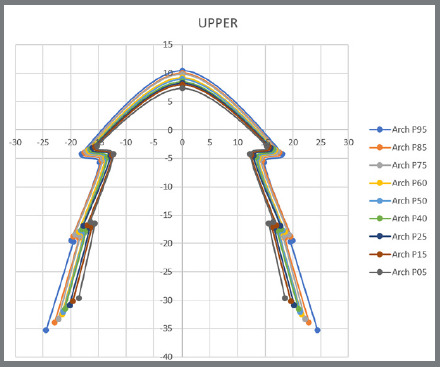
Illustration of the archwires’ points and the percentiles of the maxilla.

These data can be observed in Tables 1 and 2, and the illustration is represented by Figures 6 and 7.

**Table 1:** Coordinates measurements of each point, to set up the template of the mandibular metallic archwire.

Arch		Pt 1	Pt 2	Pt 3	Pt 4	Pt 5	Pt 6	Pt 7	Pt 8	Pt 9	Pt 10	Pt 11
P95	x	-21.6	-17.1	-18.0	-13.8	-14.9	0.0	14.9	13.8	18.0	17.1	21.6
	y	-34.3	-19.0	-18.7	-4.4	-3.7	6.9	-3.7	-4.4	-18.7	-19.0	-34.3
P85	x	-21.0	-16.6	-17.4	-13.2	-14.3	0.0	14.3	13.2	17.4	16.6	21.0
	y	-33.1	-18.4	-18.1	-4.3	-3.2	6.3	-3.2	-4.3	-18.1	-18.4	-33.1
P75	x	-20.3	-16.1	-17.0	-12.8	-14.0	0.0	14.0	12.8	17.0	16.1	20.3
	y	-32.2	-17.9	-17.7	-4.1	-3.1	6.0	-3.1	-4.1	-17.7	-17.9	-32.2
P60	x	-19.8	-15.6	-16.6	-12.5	-13.7	0.0	13.7	12.5	16.6	15.6	19.8
	y	-31.6	-17.6	-17.2	-3.8	-2.9	5.7	-2.9	-3.8	-17.2	-17.6	-31.6
P50	x	-19.5	-15.3	-16.1	-12.2	-13.6	0.0	13.6	12.2	16.1	15.3	19.5
	y	-31.2	-17.3	-17.0	-3.7	-2.8	5.3	-2.8	-3.7	-17.0	-17.3	-31.2
P40	x	-19.2	-15.0	-15.7	-11.9	-13.4	0.0	13.4	11.9	15.7	15.0	19.2
	y	-30.9	-17.1	-16.7	-3.6	-2.7	5.1	-2.7	-3.6	-16.7	-17.1	-30.9
P25	x	-18.6	-14.6	-15.3	-11.6	-13.2	0.0	13.2	11.6	15.3	14.6	18.6
	y	-30.0	-16.8	-16.4	-3.3	-2.5	4.8	-2.5	-3.3	-16.4	-16.8	-30.0
P15	x	-18.4	-14.4	-15.2	-11.3	-12.9	0.0	12.9	11.3	15.2	14.4	18.4
	y	-29.6	-16.5	-16.0	-3.2	-2.3	4.6	-2.3	-3.2	-16.0	-16.5	-29.6
P05	x	-17.6	-13.5	-14.7	-11.0	-12.4	0.0	12.4	11.0	14.7	13.5	17.6
	y	-29.1	-15.7	-15.1	-2.9	-2.2	4.2	-2.2	-2.9	-15.1	-15.7	-29.1

**Table 2:** Coordinates measurements of each point, to set up the template of the maxillary metallic archwire.

Arch		Pt 1	Pt 2	Pt 3	Pt 4	Pt 5	Pt 6	Pt 7	Pt 8	Pt 9	Pt 10	Pt 11
P95	x	-24.4	-19.5	-19.9	-14.8	-18.1	0.0	18.1	14.8	19.9	19.5	24.4
	y	-35.3	-19.7	-19.5	-5.7	-4.2	10.5	-4.2	-5.7	-19.5	-19.7	-35.3
P85	x	-22.9	-18.5	-19.5	-14.5	-17.5	0.0	17.5	14.5	19.5	18.5	22.9
	y	-33.9	-19.1	-18.8	-5.4	-3.9	10.0	-3.9	-5.4	-18.8	-19.1	-33.9
P75	x	-22.2	-18.3	-19.2	-14.2	-17.3	0.0	17.3	14.2	19.2	18.3	22.2
	y	-33.3	-18.7	-18.5	-5.1	-3.7	9.8	-3.7	-5.1	-18.5	-18.7	-33.3
P60	x	-21.5	-17.6	-18.5	-13.8	-16.8	0.0	16.8	13.8	18.5	17.6	21.5
	y	-32.5	-18.3	-18.0	-5.0	-3.5	9.2	-3.5	-5.0	-18.0	-18.3	-32.5
P50	x	-21.3	-17.5	-18.1	-13.5	-16.5	0.0	16.5	13.5	18.1	17.5	21.3
	y	-32.0	-18.1	-17.7	-4.9	-3.4	9.0	-3.4	-4.9	-17.7	-18.1	-32.0
P40	x	-21.0	-17.2	-17.9	-13.3	-16.3	0.0	16.3	13.3	17.9	17.2	21.0
	y	-31.5	-17.7	-17.4	-4.8	-3.3	8.5	-3.3	-4.8	-17.4	-17.7	-31.5
P25	x	-20.2	-16.6	-17.7	-12.8	-16.0	0.0	16.0	12.8	17.7	16.6	20.2
	y	-30.9	-17.3	-16.9	-4.6	-3.1	8.2	-3.1	-4.6	-16.9	-17.3	-30.9
P15	x	-19.6	-16.2	-17.0	-12.5	-15.6	0.0	15.6	12.5	17.0	16.2	19.6
	y	-30.2	-17.1	-16.8	-4.4	-2.9	8.0	-2.9	-4.4	-16.8	-17.1	-30.2
P05	x	-18.5	-15.6	-16.4	-12.2	-15.2	0.0	15.2	12.2	16.4	15.6	18.5
	y	-29.7	-16.5	-16.2	-4.2	-2.8	7.3	-2.8	-4.2	-16.2	-16.5	-29.7

**Figure 8:**
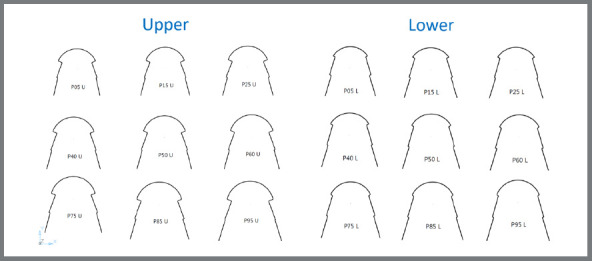
Template with eighteen anatomic lingual archwires.

After completing all these steps, 0.5 mm was deducted to compensate for the base of the lingual bracket, and the final result with the template of anatomical lingual archwires can be seen in Figure 8.

### DATA ANALYSIS AND RESULTS

In the distribution of the models analyzed in the sample, 40% were from male and 60%, from female patients. The Shapiro-Wilk test showed that the data had a normal distribution and that all measurements passed the criterion of normality. Student’s *t*-test was used to compare differences between the genders and to define the measurements. As seven measurements were made in each arch, to maintain the global significance level at 0.05, Bonferroni correction (0.05/7) was used, so the difference was considered statistically significant when *p*< 0.007.

Method error was performed after 30 days, and we repeated setting the landmarks in 30% of the sample. The Intraclass Coefficient Correlation (ICC) showed that the measurements varied from 0.98 to 1.00, and the Bland-Altman plot showed that the largest mean variation was 0.08 mm (-0.06 to 0.23), which occurred in the measurement of the second left molars.

Table 3 shows the comparison between the means of vertical and horizontal linear measurements between the genders. In the maxilla, we observed differences between the genders in molars, premolars and canines, whereas in the mandible, differences were observed only in the measurements of the molars and premolars.

**Table 3:** Comparison between the male and female genders (mm)

Measurement		Female		Male		diff.	p
		mean	SD	mean	SD		
Mandible	CI	5.05	0.85	5.29	0.78	0.24	0.241
	LI	3.56	0.62	3.73	0.56	0.17	0.248
	C	23.20	1.17	23.71	1.28	0.51	0.090
	PM1	26.44	1.61	27.27	1.66	0.83	0.041*
	PM2	29.96	1.93	30.88	1.70	0.91	0.046*
	M1	32.27	2.18	33.60	1.81	1.33	0.010*
	M2	38.42	2.45	39.89	2.07	1.47	0.011*
Maxilla	CI	8.38	1.00	8.53	0.97	0.15	0.531
	LI	5.39	0.58	5.52	0.71	0.12	0.436
	C	29.90	1.43	31.28	1.49	1.38	<0.001*
	PM1	28.74	1.69	29.95	1.64	1.21	0.004*
	PM2	33.77	2.10	35.23	1.91	1.45	0.004*
	M1	36.37	2.38	38.35	1.96	1.98	0.001*
	M2	41.67	2.84	43.70	2.57	2.02	0.003*

* Statistically significant difference p<0.007. CI = central incisor, LI = lateral incisor, C = canine, PM1 = first premolar, PM2 = second premolar, M1 = first molar, M2 = second molar.

To define the measures of the average arch, the mean was used: the 25th percentile (P25%) was used for the small arch and the 75th percentile (P75%) was used for the large arch for both males (Table 4) and females (Table 5).

**Table 4:** Measurements for the male gender (mm).

Measurement		Mean	SD	median	minimum	maximum	P25%	P75%
Mandible	CI	5.3	0.8	5.4	3.3	6.8	4.7	5.9
	LI	3.7	0.6	3.8	2.0	4.6	3.3	4.2
	C	23.7	1.3	23.8	21.1	25.7	22.7	24.6
	PM1	27.3	1.7	27.2	24.0	30.2	26.4	28.3
	PM2	30.9	1.7	31.1	27.5	34.2	29.5	31.9
	M1	33.6	1.8	33.6	30.3	37.4	31.9	34.9
	M2	39.9	2.1	40.0	35.4	42.8	38.1	42.1
Maxilla	CI	8.5	1.0	8.5	6.2	10.6	8.1	9.2
	LI	5.5	0.7	5.5	4.3	7.0	4.8	6.1
	C	31.3	1.5	31.5	28.9	35.3	30.1	31.9
	PM1	30.0	1.6	30.0	27.1	33.7	28.7	31.2
	PM2	35.2	1.9	35.2	32.0	39.6	33.6	36.5
	M1	38.4	2.0	38.2	34.1	41.8	37.0	40.0
	M2	43.7	2.6	43.4	36.6	48.7	42.4	45.7

SD= standard deviation. CI = central incisor, LI = lateral incisor, C = canine, PM1 = first premolar, PM2 = second premolar, M1 = first molar, M2 = second molar.

**Table 5:** Measurements for the female gender (mm).

Measurement		Mean	SD	median	minimum	maximum	P25%	P75%
Mandible	CI	5.1	0.9	4.9	3.7	7.3	4.5	5.5
	LI	3.6	0.6	3.6	2.5	5.1	3.1	4.0
	C	23.2	1.2	23.1	21.2	25.9	22.6	23.7
	PM1	26.4	1.6	26.2	22.8	29.9	25.2	27.2
	PM2	30.0	1.9	29.5	25.5	34.5	28.8	31.7
	M1	32.3	2.2	32.2	28.1	36.3	31.1	33.6
	M2	38.4	2.4	38.4	33.6	43.9	36.8	39.9
Maxilla	CI	8.4	1.0	8.2	6.8	10.4	7.5	9.2
	LI	5.4	0.6	5.3	3.6	6.4	5.1	5.6
	C	29.9	1.4	29.7	27.4	33.1	28.9	30.8
	PM1	28.7	1.7	28.5	25.6	32.2	27.4	30.3
	PM2	33.8	2.1	33.5	29.5	38.2	32.2	35.7
	M1	36.4	2.4	36.3	32.0	42.2	34.9	37.4
	M2	41.7	2.8	42.5	35.5	49.0	39.2	43.7

**Figure 9:**
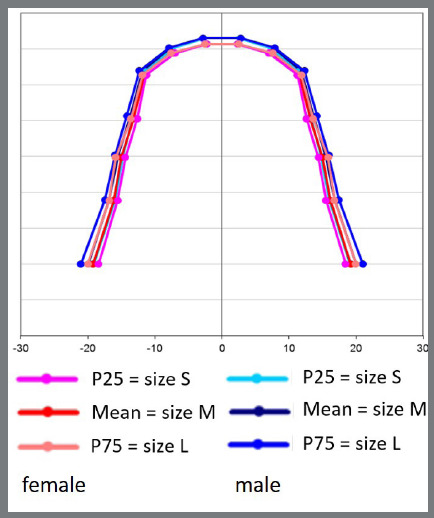
Shape and sizes of mandibular dental arches for both genders.

**Figure 10:**
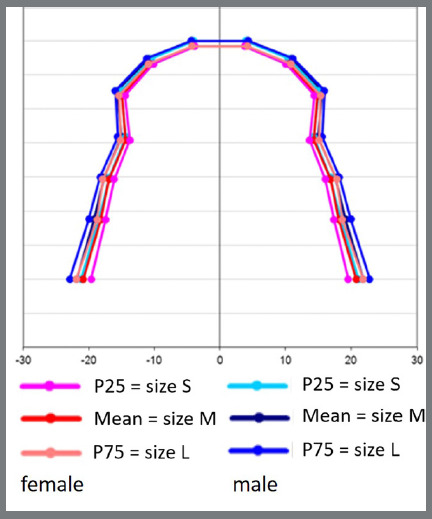
Shapes and sizes of maxillary dental arches for both genders.

Figures 9 and 10 illustrate the shapes and sizes of the arches of the two genders, for the mandible and maxilla, respectively, from the values of Tables 4 and 5. P25% values were considered small size (S), P75% values were considered large size (L), and means were used to define medium size (M). The determination of S, M and L was done separately for each gender, with pink representing the female gender and blue representing the male gender, showing twelve sizes of dental arches, six for the maxilla and six for the mandible.

Figure 8 illustrates the template with the design of eighteen archwires, with nine maxillary and nine mandibular lingual archwires.

## DISCUSSION

This study aimed at identifying the shapes and sizes of lingual dental arches from digitized models, as well as the probable difference between genders, from the point of view of the lingual orthodontic technique. It was found that there was a difference between the lingual sizes of the maxillary and mandibular arches, which allowed to define six sizes of lingual dental arches for the maxilla and six for mandible. Three of those for females and three for males, described as S, M and L.

Another purpose was to create arch templates to assist orthodontists in bending archwires themselves and not to depend on prefabricated archwires that might not meet their needs in daily practice. It was possible to create a template composed of eighteen archwire sizes, nine for the maxilla and nine for the mandible.

3D images digitized from cast models were used, in agreement with other authors,^15^ ^,^ ^24^ ^-^ ^29^ because they allow simultaneous visualization in three dimensions (horizontal, sagittal and vertical). 3D technology is currently used in several areas of Dentistry,^30,31^ allowing professionals to develop studies using the same sample. Since it can be inserted in any computer compatible with the program that was used, it is maintained over time and does not occupy a physical space, in addition to the great accuracy given by the software that is needed when using several pieces of numerical data.

One of the limitations of this study was the average age of 16.4 years, because the cast models available for digitization came from a sample of individuals aged 15 to 21.3 years old. However, this average age allows consideration of individuals without growth, since the dental arch has greater growth from 12 to 15 years old, showing a small reduction in width and depth between the ages of 15 and 26 years in both arches.^32^

To guarantee that the measurements remained proportional, regardless of the position of the digital models, the X, Y and Z axes were used in this study, as well as some previous studies,^15^ ^,^ ^24^ allowing the reference points to be positioned in the three dimensions. However, some other studies^13^ ^,^ ^14^ used only two (X and Y) coordinates, not allowing the models to be moved due to the lack of a third axis, since the Z axis does not exist in 2D models.

Fourteen points were chosen on the lingual surfaces because they would represent the place where the brackets would be placed on the lingual surfaces of the teeth, and where the orthodontic wires or metallic archwires pass into the slot of these brackets. The height chosen for these points took into consideration the most concave and convex parts of the lingual surfaces, to represent the anatomical shape of the dental arches as much as possible. From these points, the measurements were obtained to define the shapes and sizes of the arches.

To accomplish this, several authors^13^ ^,^ ^14^ ^,^ ^27^ ^,^ ^33^ used polynomial functions; others chose linear measurements.^12^ ^,^ ^15^ In this study, fourteen linear measurements (ten horizontal and four vertical) were used to define the shapes and sizes of the dental arches, similar to an earlier study,^15^ while others used four linear measurements^12^ (two horizontal and two vertical). Few studies combined the two forms together, that is, polynomial functions and linear measurements: ten linear measurements (five horizontal and five vertical),^7^ six horizontal linear measurements,^10^ and six linear measurements (three horizontal and three vertical).^34^

The linear measurements of this study were automatically defined by Delcam Power SHAPE™ 2010 software and therefore they were obtained with great precision. With this method, using methodology similar to an earlier study,^15^ we noticed a difference between the measurements when points were used more at the center of the clinical crown than when these points were positioned more in the cervical region of the clinical crown. Therefore, as shown in Table 4, the male measurements of the vertical distances of the mandibular central incisors (CI, 5.3 mm), mandibular lateral incisors (LI, 3.7 mm) and horizontal distances of mandibular canines (C, 23.7 mm) were different from those of a previous study^15^ (4.7 mm for CI, 3.3 mm for LI and 22.7 mm for C). The measurements of the distances for the maxillary central incisors (CI, 8.5 mm) and maxillary canines (C, 31.3 mm) were also different when compared with the previous report (7.5 mm for CI and 29.2 mm for C).

The same was observed with the results obtained from female samples, as shown in Table 5. It was shown that the measurements of the vertical distances of the mandibular central incisors (CI, 5.1 mm), mandibular lateral incisors (LI, 3.6 mm) and horizontal distances of the mandibular canines (C, 23.2 mm) were different from those of the previous study^15^ (4.6 mm for CI, 3.1 mm for LI, and 22.1 mm for C), The measurements of the distances for the maxillary central incisors (CI, 8.4 mm) and maxillary canines (C 29.9 mm) were also different when compared with the previous report (7.3 mm for CI and 27.9 mm for C). The differences that were found in the distance measurements are important because they change the final shape of the dental arches.

The accuracy of the digital technique was confirmed by the usage of the Shapiro-Wilk test on the measurements obtained. And as all data passed the normality criterion, even though the sample pool was composed by 40% male and 60% female models, it was possible to apply Student’s *t* test and Bonferroni test on the measures found, allowing the creation of two tables (Tables 4 and 5), showing the measures of the female and male casts separately.

Some studies^13,14,27,28,35^ were not able to identify differences between the genders. It is anthropologically known that the sizes of the male dental arches are larger than those of females,^24^ although in this study there were females with broader arches and males with narrow arches. This can be explained by the choice of reference points and the number of measurements applied.^15^

From the measurements obtained, three sizes of dental arches were identified: S, M, and L. In another study,^14^ median measurements were applied, unlike the averages found in this study and those of other authors.^15^ In this way, the mean, and not the median, was used to obtain the final measurements, because mean measurements were more accurate than the medians.

In the mandible, the shape of the dental arch resembles a parabola, with a more rounded form in the anterior portion, and the posterior side is more like a straight line, with a slight deviation in the region of the premolars and molars. In the maxilla, the shape of the dental arch also resembles a parabola, with the rounded anterior portion with more pronounced curves in the canine region, and a segment of a line, with deviations, in the region of the premolars and molars (Figs 9 and 10). In other studies,^14^ ^,^ ^15^ the parabola was flatter, with less prominent curve in the anterior region, mainly due to the location of the points that were selected. In our study, those points were placed more occlusally, to allow a better adaptation to the anatomical form of the dental arch, compared to previous studies in which the placement of the points was more cervical. 

As stated in a previous study,^36^ to perform orthodontic treatment orthodontist should have an understanding about the shape of the dental arch. It has been observed for years that many professionals sought to find a method to reliably copy the shape of the dental arch and apply it for orthodontic treatment.

For this purpose, the first diagram in orthodontics was created by the millimeter paper method.^37^ Over the years, other diagrams have emerged,^38^ ^-^ ^41^ with the intention of preserving stability and individualizing treatment, allowing coordination of archwires to facilitate the professional’s work. Some are more accurate and use their own initial model as a reference;^36^ ^,^ ^38^ others look for diagrams with the possibility of several sizes of archwires,^40,41^ which is possible in regard to making metal archwires.

Some studies^42,43^ have shown that professionals have a concern regarding maintenance of the dental arch shape. Perhaps because the prefabricated archwires mostly do not correspond to the size and shape of the arches in a normal occlusion.^26^ Nevertheless, it should be noted that prefabricated light alloy archwires (mainly nickel and titanium) cannot substantially alter the shape of the dental arch;^25^ however, they assist in the initial stages of treatment and are necessary for current orthodontics.^26^

In lingual orthodontics, diagrams were proposed from photocopied models,^6^ and others through computerized programs,^9^ ^,^ ^14^ ^,^ ^15^ ^,^ ^44^ to allow the manufacture of individualized orthodontic archwires, because in lingual orthodontics the coordination of the archwires is a difficult or almost impossible task.

Therefore, considering that dental human arches are asymmetrical and that this characteristic is more a rule than an exception,^45^ the construction of symmetrical archwires results in smaller errors than if the asymmetries are obeyed,^39^ and the measures resulting from this work are adequate to define the shapes of anatomical arches. It is possible to determine a diagram to obtain lingual archwires that could help lingual orthodontics in the definition of prefabricated archwires.

There is controversy regarding the various types of archwires used in the lingual technique. Some authors^14^ ^-^ ^18^ suggest using straight archwire as a facilitator of the technique, even aware of the need for making bends for finishing and detailing. A group of authors^1^ ^,^ ^2^ ^,^ ^3^ have advocated the lingual technique using mushroom archwires, and others^6^ advocated metallic archwires to be used in the lingual technique resembling a Christmas tree-shaped archwire.

It is known that there are differences between one archwire type and another, and it is important to remember that in clinical practice, the mechanical aspect must also be considered. Therefore, authors who compared *in vitro* the two types of mushroom and straight archwire observed that the advantages and disadvantages of some orthodontic movements varied for each archwire according to the treatment phase.^19^ ^,^ ^20^

We believe that more studies on archwire shape should be performed (*in vitro* and *in vivo*) because it is still not possible to state which archwire shapes -mushroom, straight wire, Christmas tree or anatomical (the forms found in this study)- will be more suitable for treatment with the lingual technique. Therefore, the professional will have the opportunity to choose the shape of the archwire that better suits according to the clinical case being treated and, if possible, a template to assist in the orthodontic treatment.

## CONCLUSION

In this study, we were able to define twelve sizes of lingual dental arches by altering the reference points: six sizes for the maxilla, with three for females (S, M, L) and three for males (S, M, L); and six sizes for the mandible, with three for females (S, M, L) and three for males (S, M, L).

It was also possible to create an anatomical template representative of anatomical arch shapes, allowing construction of lingual metallic archwires to be used in the lingual technique that are more compatible with the reality of the anatomy of the dental arches.
